# The role of miR-122 in the dysregulation of glucose-6-phosphate dehydrogenase (G6PD) expression in hepatocellular cancer

**DOI:** 10.1038/s41598-018-27358-5

**Published:** 2018-06-14

**Authors:** Juan M. Barajas, Ryan Reyes, Maria J. Guerrero, Samson T. Jacob, Tasneem Motiwala, Kalpana Ghoshal

**Affiliations:** 1 Department of Pathology, The Ohio State University, Columbus, OH 43210 USA; 2 Department of Cancer Biology and Genetics, The Ohio State University, Columbus, OH 43210 USA; 3 Department of Biomedical Informatics, The Ohio State University, Columbus, OH 43210 USA; 4 Comprehensive Cancer Center, The Ohio State University, Columbus, OH 43210 USA

## Abstract

Hepatocellular carcinoma (HCC) is the second leading cause of cancer-related deaths worldwide. Thus, a better understanding of molecular aberrations involved in HCC pathogenesis is necessary for developing effective therapy. It is well established that cancer cells metabolize energy sources differently to rapidly generate biomass. Glucose-6-phosphate-dehydrogenase (G6PD), the rate-limiting enzyme of the Pentose Phosphate Pathway (PPP), is often activated in human malignancies to generate precursors for nucleotide and lipid synthesis. Here, we determined the clinical significance of G6PD in primary human HCC by analyzing RNA-seq and clinical data in The Cancer Genome Atlas. We found that the upregulation of G6PD correlates with higher tumor grade, increased tumor recurrence, and poor patient survival. Notably, liver-specific miR-122, which is essential for metabolic homeostasis, suppresses G6PD expression by directly interacting with its 3′UTR. Luciferase reporter assay confirmed two conserved functional miR-122 binding sites located in the 3′-UTR of G6PD. Furthermore, we show that ectopic expression of miR-122 and miR-1, a known regulator of G6PD expression coordinately repress G6PD expression in HCC cells. These miRNAs also reduced G6PD activity in HepG2 cells that express relatively high activity of this enzyme. Collectively, this study provides evidence that anti-HCC efficacy of miR122 and miR-1 could be mediated, at least in part, through inhibition of PPP by suppressing the expression of G6PD.

## Introduction

Hepatocellular carcinoma (HCC) is the second-deadliest malignancy worldwide^1^. Liver tumorigenesis is a multi-step process typically arising from persistent liver damage caused by an underlying liver disease such as alcohol consumption, HCV/HBV viral infection, or metabolic syndrome^2^. Hepatocellular carcinoma (HCC) is the most common primary liver cancer with limited treatment options such as sorafenib^3^, nivolumab^4^, and regorafenib^5^ for patients with advanced disease. Therefore, it is critical to have a better molecular and metabolic understanding of HCC initiation and progression to improve therapeutic discovery and reduction in overall tumor burden.

Glucose-6-Phosphate-Dehydrogenase (G6PD) is the rate-limiting enzyme of the pentose phosphate pathway (PPP). It generates NADPH required for lipid synthesis and generation of ribose phosphates which are precursors for nucleotide synthesis. Redirecting the carbon flux to the PPP allows these cancer cells to generate biomass and proliferate rapidly^6–9^, an essential step in tumorigenesis. Interest in therapeutic discovery by targeting PPP has increased over the last several years in several human malignancies^10,11^. Notably, G6PD activity and expression are elevated by oncogenes such as K-RAS^12^, suppressed by tumor suppressors like P53^13^ or PTEN^14^, and correlate with poor survival in the context of ID1 expression^11^. However, G6PD regulation is still not completely understood in hepatocellular carcinoma.

Several groups including ours demonstrated that miR-122, a highly conserved liver-specific miRNA expressed in vertebrates^15^, is a novel tumor suppressor in HCC^16–18^. This small (21–23 nt) non-coding RNA regulates gene expression post-transcriptionally by mediating Argonaute binding at the target RNA sites complementary to the miR-122 seed sequence, causing decay of target message^19^. Studies have shown that miR-122 maintains liver homeostasis by regulating triglyceride and cholesterol metabolism^20,21^, mitochondrial function^22^, expression of genes modulated by circadian rhythm^23^, polyploidy^24^, and tumor suppression^16,17,25–30^. While the role of miR-122 in lipid metabolism has been studied^16,17,20,31^, our understanding of its potential role in glucose metabolism remains unclear^32^. Interestingly, G6PD was validated as a miR-1 target^33,34^, indicating that complex networks of miRNA interactions may regulate G6PD.

Here, we demonstrate that G6PD levels are altered in liver cancer patients from The Cancer Genome Atlas (http://cancergenome.nih.gov/), its mRNA levels increase in conjunction with rise tumor grade, and its levels also negatively correlate with the expression of miR-122 and miR-1, a previously reported regulator of G6PD^33,34^. We have also shown that G6PD harbors three miR-122 binding sites in its 3′UTR region, and validated two of the conserved sites using luciferase reporter assay. Exogenous miR-122 and miR-1 mimics resulted in reduced G6PD expression and activity in transfected HCC cells. To the best of our knowledge, these data demonstrate for the first time that miR-122 regulates G6PD levels in HCC cells, and that loss of expression of miR-1 and miR-122 in primary HCCs may contribute to the increased G6PD activity thereby promoting tumor growth.

## Results

### Upregulation of G6PD expression is associated with poor prognosis in human hepatocellular carcinoma patients

**Figure 1:**
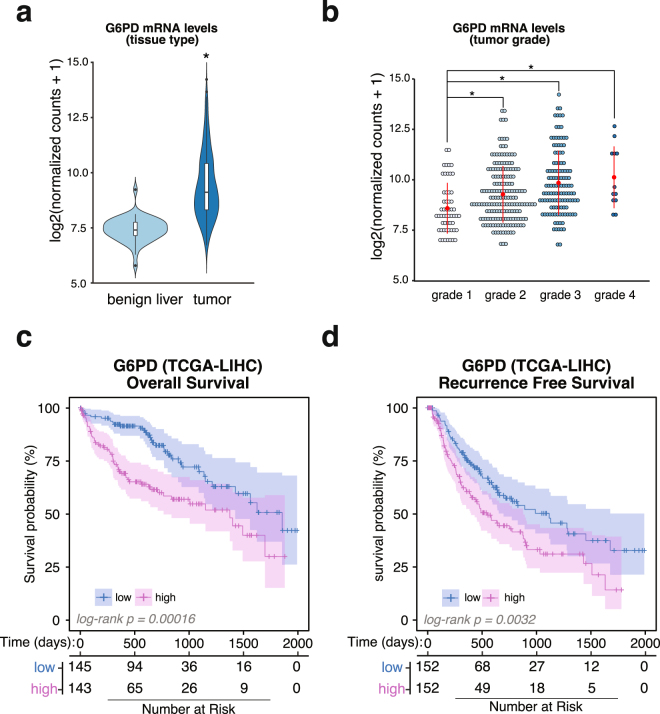
G6PD is altered in human HCC. (**a**) G6PD mRNA levels in tumors from liver cancer patients. Normalized mRNA levels from benign livers (n = 50) and liver tumors (n = 377) quantified using RNA-seq were queried from The Cancer Genome Atlas. Significance was determined using Welch’s T-Test function in R (p-value = 1.082346e-40). **(b)** Dot-plot of G6PD mRNA levels in different tumor grades (ANOVA, P-value = 6.79e-07). Comparison between means using Tukey Honest Significance Test in R revealed significant difference in G6PD mRNA levels with increasing tumor grade (Supplementary Table 1). **(c)** Kaplan-Meier curve of G6PD overall survival in liver cancer patients (n = 318, patients with missing data or surviving past 5 years were removed from the analysis). Patients were stratified by high expression (top 50th percentile), and low expression (bottom 50th percentile). **(d)** Kaplan-Meier curve of tumor recurrence in liver cancer patients with respect to G6PD levels. Analysis was done as described in **(c)**.

To analyze the expression profile of G6PD in liver cancer, we queried Liver and Hepatocellular Cancer (LIHC) RNA-seq data from The Cancer Genome Atlas (TCGA) database using the Xena Cancer Browser (xena.ucsc.edu; xenabrowser.net). We found that G6PD mRNA levels are significantly upregulated in primary tumors from human HCC patients (P-value = 1.082346 × 10^−40^) when compared to benign tissue (Fig. 1a). Furthermore, patient samples organized by tumor grade displayed a progressive increase in G6PD mRNA levels as determined using ANOVA (ANOVA, P-value = 6.79 × 10^−7^), indicating a strong association with tumor progression (Fig. 1b). Multi-comparison analysis revealed a significant difference in G6PD expression among progressing tumor grades (Supplement Table 1). We also found that increased levels of G6PD mRNA were associated with a reduced patient outcome (Log-Rank P-value = 0.00015) (Fig. 1c) and increased tumor recurrence (Log-Rank P-value = 0.0032) (Fig. 1d). G6PD protein and mRNA levels were measured in the extracts prepared from frozen primary human HCC and benign liver samples procured from the Cooperative Human Tissue Network (Supplement Fig. 1a,b). G6PD protein and mRNA levels were upregulated in 3 and 6 tumors relative to the pair-matched liver tissues in 8 specimens analyzed, suggesting that G6PD mRNA and protein levels were not coordinately regulated in these primary HCCs analyzed.

### G6PD is a novel conserved miR-122 target

**Figure 2:**
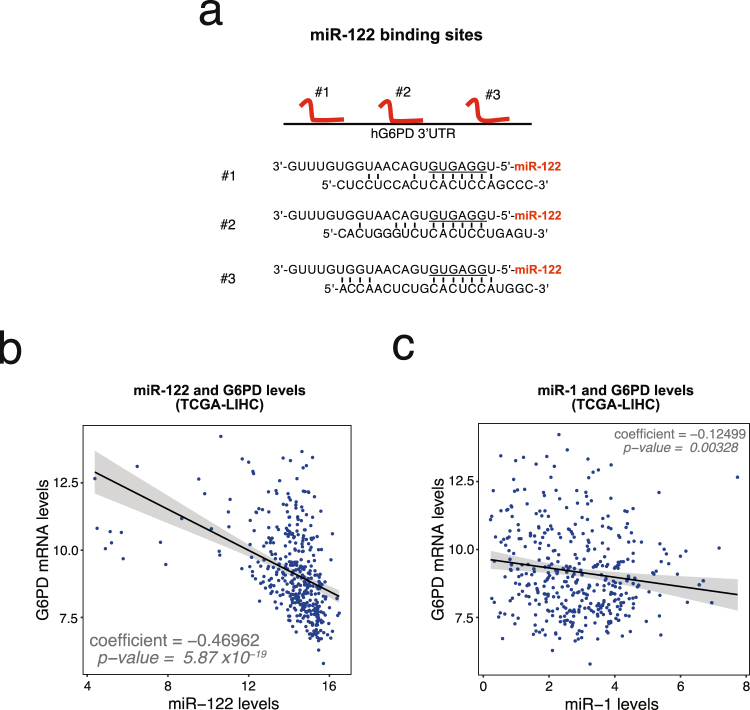
G6PD is a novel target of miR-122. (**a)** Schematic depicting miR-122 sites located on the human G6PD 3′-UTR. **(b**,**c)** G6PD normalized expression (log2(normalized count +1)) and RSEM expression (log2) of miR-122 **(b)** and miR-1 **(c)** were plotted per tumor sample. miRNA and mRNA expression data in liver cancer patients was queried from The Cancer Genome Atlas using Xena UCSC Browser (xena.ucsc.edu; xenabrowser.net). Regression coefficients and corresponding p-values are shown.

**Figure 3:**
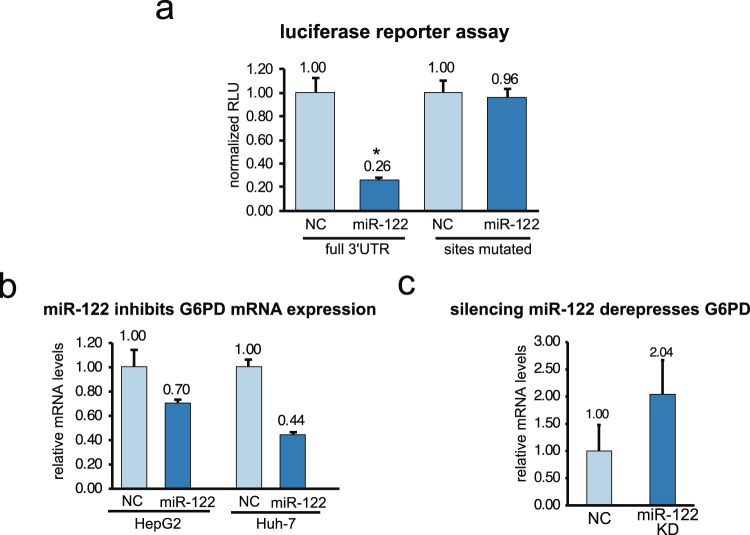
Validation of G6PD as a novel miR-122 target. **(a)** Luciferase reporter assay of G6PD 3′-UTR (wild type or mutant) driven *Renilla* luciferase activity normalized to firefly luciferase after transfection H293T cells with scrambled (NC) or miR-122 mimic RNA (miR-122). **(b**,**c)** G6PD mRNA levels in HCC cells transfected with in miR-122 mimic or scrambled (NC) RNA (**b**), and in Huh-7 cells transfected with miR-122 anti-sense oligo (miR-122 KD) or negative control oligo (NC) (**c**). mRNA levels were measured by RT-qPCR.

A large number of studies have shown that miRNAs are important regulators of glucose metabolism in HCC^35^. A query of the miRNA target prediction database TargetScan^36^ and Ago-CLIP data performed in human benign liver and liver tumors (GSE97061)^30^, revealed that G6PD harbors three miR-122 binding sites in the 3′UTR (Fig. 2a). G6PD mRNA and miR-122 levels in liver cancer patient data downloaded from TCGA were found to negatively correlate (Fig. 2b) (R-squared = 0.1772, P-value = 5.874367 × 10^−19^, regression coefficient = −0.4696232). In contrast, the correlation of G6PD and miR-1 expression was less pronounced (Fig. 2c) (R-squared = 0.01891, P-value = 0.003279, regression coefficient = −0.1249872). Both miR-1 and miR-122 were found to be suppressed in tumor when compared benign liver tissues (Supplement Fig. 2). Luciferase reporter assay revealed functional repression of *Renilla* luciferase harboring the full-length G6PD 3′-UTR by miR-122 (Fig. 3a). Complete recovery of this activity in cells co-transfected with luciferase reporter plasmid harboring the G6PD 3′-UTR with mutated miR-122 binding sites confirmed that G6PD 3′-UTR is required for miR-122 mediated suppression (Fig. 3a). Mutation (point or deletion) of individual sites showed that miR-122 cognate sites #2 and #3 are functional (Supplement Fig. 3a,b). As reported earlier^33,34^, we also found that miR-1 targets G6PD by interacting with a cognate site in its 3′-UTR (Supplement Fig. 3c,d). Furthermore, G6PD mRNA levels was significantly reduced in HCC cells transfected with miR-122 mimics (Fig. 3b) whereas knocking down miR-122 by transfecting an antimiR-122 oligo resulted in increased G6PD mRNA levels (Fig. 3c).

### miR-122 and miR-1 downregulate G6PD expression in liver cancer cells individually and in combination

**Figure 4:**
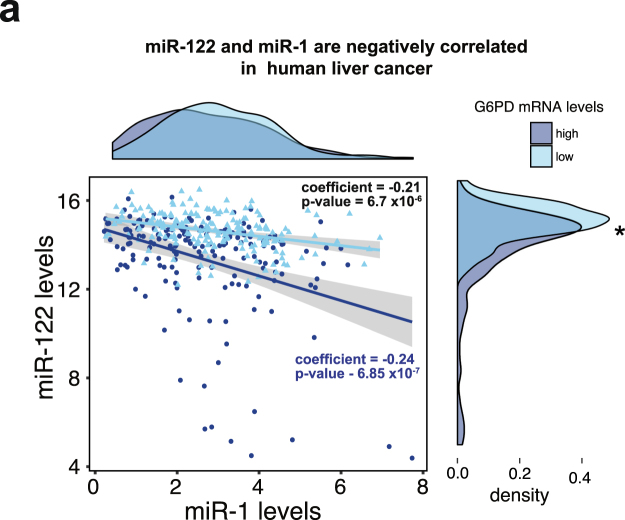
miR-1 and miR-122 levels are negatively correlated in human liver cancer. Scatterplot and linear regression of miR-122 and miR-1 levels in liver cancer patients. The LIHC-TCGA data downloaded from UCSC Xena Cancer browser (xenabrowser.net) was plotted and linear regression coefficients were calculated using the lm() function in R. Overall regression coefficient between miR-122 and miR-1 was calculated as −0.212 (p-value = 1.22 × 107). Data was further stratified by G6PD mRNA levels (high = dark blue, low = light blue). Individual coefficients and p-values were calculated for each condition using the lm() function in R and are shown on the plot. Density plots were used to visualize the distribution of miR-1 (top panel) and miR-122 (right panel) levels in the context of G6PD mRNA levels. Kolmogorov–Smirnov test was used to calculate significant differences in the relative distribution of miRNA and G6PD mRNA levels (miR-122 p-value = 1.94 × 10^−8^, miR-1 p-value = 0.09263).

**Figure 5:**
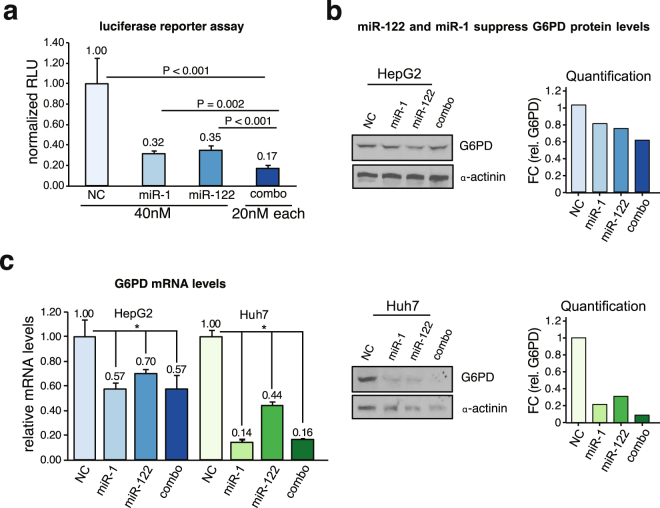
miR-1 and miR-122 reppress G6PD expression by direct targeting. (**a**) G6PD was validated as a miR-1 and miR-122 target using luciferase reporter assay. H293-T cells were transfected with control RNA (NC), miR-122, miR-1, or combination of both, along with psi-CHECK2 vector harboring human G6PD 3′-UTR and Firefly luciferase (an internal control). After 48 hours, luciferase activities were measured in cell extracts per manufacture’s protocol (Promega). **(b**,**c)** G6PD immunoblot and RT-qPCR analysis shows depletion of G6PD protein level (**b**) and mRNA level (**c**) in HCC cells transfected with miRNA mimics (50 nM) for 48hrs. (* indicates p-value < 0.05, p-value was calculated using Student T-test). Cropped immunoblot images in (**b**) were obtained from the same gel. Full immunoblot images are found in supplemental material.

Previous studies have shown that miR-1 levels are reduced in several malignancies, and it directly regulates G6PD expression by binding to the G6PD 3′-UTR at a single site^33,34^. In healthy liver however, miR-122 is the most abundant miRNA, and its downregulation is much more pronounced than miR-1 in liver cancer (Supplement Fig. 2). Interestingly, levels of miR-1 and miR-122 negatively correlate with each other (overall p-value = 1.22 × 10^−7^, coefficient = −0.212) in human liver cancer (Fig. 4), indicating a possible reciprocal regulation. To address whether both could contribute equally to the upregulation of G6PD expression in liver cancer, we compared miR-1 and miR-122 levels in the context of G6PD mRNA levels using density distribution plots (Fig. 4). The density plots of miR-1 and miR-122 showed that higher levels of G6PD were associated with lower levels of miR-122. No such relationship was identified for miR-1. Nevertheless, our results showed that miR-1 and miR-122 are both capable of suppressing G6PD luciferase reporter activity independently or in combination (Fig. 5a). Interestingly, miR-1, miR-122 combination seemed to have an additive effect on luciferase activity suppression. This is also reflected at the G6PD protein and RNA levels in liver cancer cells transfected with miR-1 or miR-122 mimics (Fig. 5b,c). Notably, miR-1 and miR-122 together exhibited slightly more suppression in G6PD protein levels compared to its mRNA levels.

### miR-1 and miR-122 inhibit G6PD activity and cell survival in HepG2 cells

**Figure 6:**
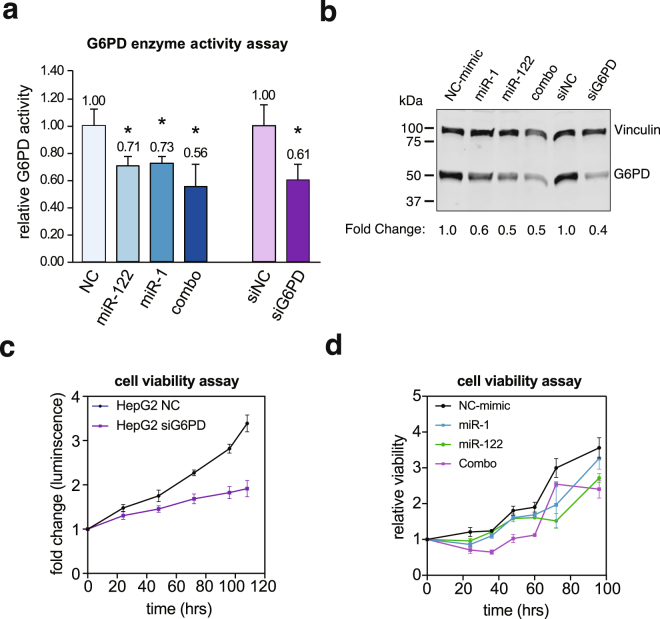
G6PD expression and activity are suppressed by miR-122 and miR-1 in HepG2 cells. HepG2 cells transfected with scrambled miRNA (NC), miR-1, miR-122, combo (miR-1 and miR-122), G6PD siRNA (siG6PD) or negative control siRNA (NC) for 48 hours. **(a)** G6PD activities in these cells were determined by measuring NADPH absorbance at 341 nm and calculating the NADP^+^/NADPH ratios. Asterisks indicates p-value < 0.05, p-value was calculated using two-tailed Student T-test). **(b)** Immunoblotting of G6PD protein was performed in the lysates. G6PD levels were normalized to that of vinculin. **(c**,**d)** Cell viability measured using CellTiter-Glo® Luminescent kit. Cell viability over time was determined as change in luminescence from 0-hour time point. Cropped immunoblot images in **(b)** were obtained from the same gel. Full immunoblot images are found in supplemental material.

Next we sought to determine whether modulation of G6PD expression by miR-1 or miR-122 is reflected in its enzyme activity. To this end, we assayed G6PD activity by measuring ratio of NADP^+^ to NADPH in Huh7 and HepG2 cells expressing relatively higher and lower miR-122 levels, respectively^37^. PPP maintains redox levels by replenishing NADPH required for lipid biosynthesis in rapidly proliferating cells through G6PD activity, and provides precursors for nucleotide and amino acid biosynthesis^38^. Indeed, the enzyme activity was much higher in the lysate of HepG2 cells compared to that of Huh7 cells and increased with increasing protein concentrations (Supplement Fig. 4). Importantly, this activity was reduced in HepG2 cells transfected with miR-1 or miR-122 mimic, which was more pronounced in cells expressing both miRNAs (Fig. 6a). Notably, the suppression of G6PD activity in miR-1 and miR-122 mimic co-transfected cells was comparable to G6PD knocked down cells. Importantly, decrease in G6PD activity correlated with reduction in protein levels in cells expressing these miRNAs alone, in combination or in siRNA - mediated G6PD - depleted cells (Fig. 6b). Since G6PD activity is essential for cell proliferation, we speculated ectopic miR-1 and miR-122 expression would inhibit HepG2 cell proliferation. Indeed, growth of these cells were suppressed upon transfecting miR-1, miR-122 and their combination relative to the scrambled miRNA (Fig. 6d). In contrast, we did not observe significant effect of G6PD depletion in Huh-7 cell survival (Supplement Fig. 5). Collectively, these data implicate anti-tumorigenic efficacy of miR-1 and miR-122 at least, in part, mediated through targeting G6PD, associated with poor patient prognosis (Fig. 1).

## Discussion

The metabolic reprogramming of cancer cells is often defined by a change to aerobic glycolysis to produce large amounts of carbon source for nucleotide, lipid, and amino acid synthesis necessary for rapid proliferation and increase cellular biomass^7,39,40^. Importantly, our lab has recently shown that targeting tumor metabolism is an effective strategy in HCC treatment^41^. G6PD is the rate-limiting enzyme of the pentose phosphate pathway that contributes to this metabolic shift by producing ribose-5-phosphate, the precursor to nucleotide synthesis, and reducing oxidative stress by replenishing NADPH reserves essential for lipid synthesis^6–9,12^. G6PD is also a prognostic marker in patients suffering from different cancers^11,14^. Analysis of liver cancer data (LIHC) from TCGA corroborates these results in liver cancer (Fig. 1). The extensive TCGA-LIHC collection of healthy, benign tissue and liver cancer samples correlated upregulation G6PD mRNA levels with higher tumor grades (Fig. 1b). These results suggest an increased dependency of poorly differentiated tumor cells on PPP for rapid biomass production. This notion is further supported by the fact that increased G6PD levels predict a worse patient prognosis and tumor recurrence in liver cancer (Fig. 1c,d). In this same dataset, we found a profound inverse correlation of miR-122 with G6PD levels in liver cancer (Fig. 2b,c), suggesting that miR-122 may play a regulatory role of PPP flux through G6PD suppression.

In the present study we established G6PD as a functional miR-122 target, and G6PD could be potential therapeutic target for HCC. Down regulation of miR-122 expression associated with or hypermethylation of upstream promoter region is predictive of poor overall survival in human liver cancer patients^18,29,37,42^. We find that the G6PD 3′-UTR harbors three miR-122 sites, and two are validated as functional sites (Fig. 3; Supplement Fig. 3). This conclusion reflects results from other studies that showed that the loss of miR-122 is associated with an altered metabolic profile in the liver^20,43,44^. Our study shows that G6PD is a critical miR-122 target that is likely to modulate glucose metabolism in liver cancer.

Previous studies have shown a notable relationship between miR-1 and G6PD^33,34^. However, our data show for the first time that levels of miR-122 are almost 10,000 fold higher than miR-1 levels in benign liver tissues in HCC patients (Supplement Fig. 2). Other similar studies have reported that level of the muscle specific miR-1 in the liver is relatively low^45^. We were one of the first to report that miR-1 is suppressed in the lung^46^ and hepatocellular cancer^47^, and its therapeutic potential in these malignancies. Inverse expression of miR-1 and G6PD in patient tissues further supports its contribution to the overall elevated G6PD levels in HCC. While the role of miR-122 depletion in HCC is much more significant due to its abundance in benign liver and its dramatic decrease in HCC, a combined reduction of both miR-122 and miR-1 are likely to contribute to the deregulation of glucose metabolism in HCC, resulting in rapid tumor progression. Furthermore, G6PD mRNA levels were more closely associated with miR-122 than with miR-1 (Fig. 4). These data also show a negative correlation between miR-1 and miR-122 indicating their reciprocal regulation. It is noteworthy that both miR-1 and miR-122 comparably suppressed luciferase activity and G6PD levels indicating both can target G6PD equally well (Fig. 4, Supplement Fig. 3). This relationship was further corroborated by the decrease in G6PD activity after miR-1 and miR-122 co-transfection in HepG2 cells (Fig. 6). Tumor suppressor functions of these two miRNAs have been amply demonstrated in diverse cancers. These data tend to support the possibility that miR-1 and miR-122 in combination could be more effective anti-HCC therapy. However, effective delivery of miRNA mimics without causing any toxicity in HCC patients is still a major challenge that needs to be overcome^48^.

## Methods

### Reagents and Antibodies

Antibodies used for western blotting were purchased from Cell Signaling (Danvers, MA, USA).

RNA mimics were obtained from Dharmacon (Lafayette, CO). All other reagents were of molecular biology grade. Catalog numbers for siRNA, antibodies, and other reagents used in this study can be found in the supplement materials (Supplement Table S2). These antibodies include the following: (G6PD, Abcam, cat# ab76598), (α-actinin, Santa Cruz, Sc-17829), (Vinculin, Protein-Tech, 66305-1-Ig), (Gapdh, Cell Signaling, 5174 L).

### Cell Lines and Culture Conditions and Transfections

Huh-7 (generously provided by Dr. James Taylor Fox Chase Center, PA) were expanded and cultured in Minimum Essential Media (MEM) (Corning, Corning, NY) supplemented with 10% fetal bovine serum (FBS, Sigma, St. Louis, MO), 0.1 mM nonessential amino acids (NEAA, Invitrogen, Carlsbad, CA) and Penicillin-Streptomycin (Corning, Corning, New York). HepG2, and H293-T (ATCC, Manassas, VA) cells were expanded and cultured in Dulbecco’s Modified Eagle Medium (DMEM) (Corning, Corning, NY) supplemented with 10% fetal bovine serum (FBS, Sigma, St. Louis, MO) and Penicillin-Streptomycin while maintaining and expanding (Corning, Corning, NY).

### RNA Isolation and RT-qPCR

Total RNA was isolated using TRIzol (Life Technologies, Carlsbad, CA). RNA was treated with DNase I (NEB, Ipswich, MA) to remove contaminant DNA and purified by phenol-chloroform extraction and ethanol precipitation. DNase treated RNA (2 μg) was reverse transcribed into complementary DNA (cDNA) using High-capacity cDNA reverse transcription kit (Applied Biosystem, Foster City, CA). Real-time quantitative PCR (RT-qPCR) was performed using SYBR Green PCR kit (ThermoFisher Scientific, Waltham, MA). The expression of G6PD was normalized to that of GAPDH/ACTB. Relative expression was calculated by comparative CT method. Primer sequences are provided in Table S2. miR-122 and miR-1 were estimated using qPCR Taqman assay kits as described^16^.

### Western blot analysis

Immunoblots were performed as previously described in our published protocol^41^. Briefly, cells prepared for immunoblot were lysed in cell lysis buffer (50 mM Tris-HCl (pH 7.5), 10 mM EDTA (pH 8.0), 1% sodium dodecyl sulfate (SDS)), supplemented with phosphatase inhibitors and PMSF). Cell lysates were fractionated by SDS - polyacrylamide (10% acrylamide) gel electrophoresis and transferred onto nitrocellulose membrane (Bio-Rad, Hercules, CA). After blocking with blocking buffer (LI-COR, Lincoln, NE) containing 0.1% Tween-20, the membrane was incubated with primary antibodies overnight at 4 °C. Following incubation with appropriate secondary antibody (IRD-680 or IRD-800) for 1 hour at room temperature, the proteins of interest were visualized using LI-COR-Odyssey infra-red scanner (LI-COR, Lincoln, NE). Protein concentrations were estimated using a Bio-Rad protein assay kit (Cat # 5000001) (Bio-Rad, Hercules, CA). Settings and full-length blots for Li-COR images are presented in Supplement Materials. For film blots, the signal was detected using an ECL Western blotting reagent (Pierce, Appleton, WI). Cropping of gels is depicted by whitespace and meant to increase clarity and conciseness. Complete unaltered images of the blots are presented in the supplement material.

### Luciferase reporter assays

Wild-type and mutated G6PD 3′-UTR harboring miR-122 and miR-1 binding sites was cloned into the 3′-UTR of *Renilla* Luciferase cDNA in psiCHECK2 (Promega, Madison, WI) dual luciferase reporter. psiCHECK2 vectors (50 ng) harboring miR-122 target 3′-UTRs were co-transfected with either miR-122, miR-1, or scrambled (NC) RNA mimics (50 nM) using Lipofectamine 3000 (Thermo Fisher Scientific, Waltham, MA) into H293-T cells. *Renilla* luciferase activity was measured after 48 hours per manufacturer’s protocol (Promega, Madison, WI) and normalized to *Firefly* luciferase activity (RLU). Mimics and scrambled RNAs for this study were purchased from Dharmacon (Dharmacon, Lafayette, CO).

### Cell viability assays

HepG2 cells seeded into a 60 mm plate (~750,000 cells/well) were allowed to grow for 24 hr. Cells were then transfected with 40 nM each of scrambled miRNA mimic control (NC-mimic), miR-1 mimic, miR-122 mimic or their combination (20 nM of each), non-targeting siRNA control (70 nM), or siG6PD (70 nM) using RNAiMAX following the manufacturer’s protocol (Thermo Fischer Scientific, Waltham, MA). Cells were allowed to recover for 12 hr and were seeded onto a 96-well strip-well plate (8 wells/strip, Corning, Corning, NY). Cells were allowed to recover for 12 hr, and CellTiter-Glo was added to first strip-well following the manufacturer’s protocol (Promega, Madison, WI) and assigned as the time point 0 hr. The luminescent supernatant was transferred to an opaque luminometer 96-well plate prior to measuring luminescence in quadruplicates. The same procedure was followed for the indicated time points and the data was normalized to 0 hr to determine fold change in cell viability.

### G6PD activity assay

G6PD activity was calculated by comparing the Glucose-6-phosphate dehydrogenase and 6-Phosphogluconate dehydrogenase (G6PD/6PGD) activity^49,50^. Briefly, HepG2 cells were transfected using Lipofectamine 3000 (Thermo Fisher Scientific, Waltham, MA) with either miR-1 (40 nM), miR-122 (40 nM), or combo (20 nM of each). After 48 hours of culture, cells were collected and lysed in non-denaturing cell lysis buffer (50 mM Tris-HCl (pH 7.5, 0.5% Triton-X-100, supplemented with phosphatase inhibitors and PMSF). Lysates (5 μg and 10 μg of protein) of each sample were added in two duplicate sets to 96-well plate. To the first condition duplicate set, we added 100 μl of Combination Reaction buffer (50 mM Tris-HCl (pH 8.1), 1 mM MgCl_2_, 0.2 mM 6PG, 0.2 mM G6P, 0.1 mM NADP^+^), and to the second duplicate set, we added 6PGD Reaction Buffer (50 mM Tris-HCl (pH 8.1), 1 mM MgCl_2_, 0.2 mM 6PG, 0.1 mM NADP^+^). Absorbance of each well was measured at 341 nm at several time-points at 37 °C. G6PD activity was calculated as the difference in absorbance (NADH levels) between the wells treated with the Combination reaction buffer and wells treated the 6PGD reaction buffer.

### Download and analysis of TCGA data

Liver hepatocellular carcinoma (LIHC) data from The Cancer Genome Atlas (TCGA) was accessed using UCSC Xena Cancer Genomics Browser (xenabrowser.net). In our data query, we included RNA-sequencing data (log2(normalized count +1)), normalized miRNA sequencing data (RSEM), and patient clinical data (tumor grade, recurrence free survival, and overall survival). Survival analysis was performed using Surv() function and regression modeling using coxph() function in R. Patients were split to groups based on the median G6PD mRNA levels, high expression (>50^th^ percentile) or low expression (<50^th^ percentile). Patients with missing values or surviving past 2000 days (~5 years) were removed from the analysis. Linear Regression modeling analysis was calculated using the linear modeling function lm() in R. RStudio running version 3.3.2 and GraphPad Prism 7 were used for all statistical analysis, modeling, and plotting. The results shown in this study are, in part, based on data generated by TCGA Research Network: cancergenome.nih.gov.

### Implications

Reprogramming of glucose metabolism is an important step in liver tumorigenesis. Pentose phosphate pathway is essential for lipid and nucleotide synthesis of rapidly proliferating cancer cells. Understanding mechanisms of microRNA mediated regulation of this pathway may lead to therapeutic benefits in patients suffering from HCC.

## Electronic supplementary material

Supplementary Material
