# Supplementary material for: The role of miR-122 in the dysregulation of glucose-6-phosphate dehydrogenase (G6PD) expression in hepatocellular cancer
Source: Sci Rep. 2018 Jun 14;8:9105. doi: 10.1038/s41598-018-27358-5 (PMC6002539; doi:10.1038/s41598-018-27358-5)
Supplement: Supplementary file 1 — Supplementary Material [file 41598_2018_27358_MOESM1_ESM.docx]

Supplemental Information

**The role of miR-122 in the dysregulation of glucose-6-phosphate dehydrogenase (G6PD) in hepatocellular cancer**

Juan M. Barajas^1,4,#^, Ryan Reyes^2,#^, Maria J. Guerrero^1,4^, Samson T. Jacob^2*^, Tasneem Motiwala^3,4*^, and Kalpana Ghoshal^1,4*^

^1^Department of Pathology, ^2^Department of Cancer Biology and Genetics, ^3^Department of Biomedical Informatics, ^4^Comprehensive Cancer Center, The Ohio State University, Columbus, OH 43210, USA

^#^These authors contributed equally to this work


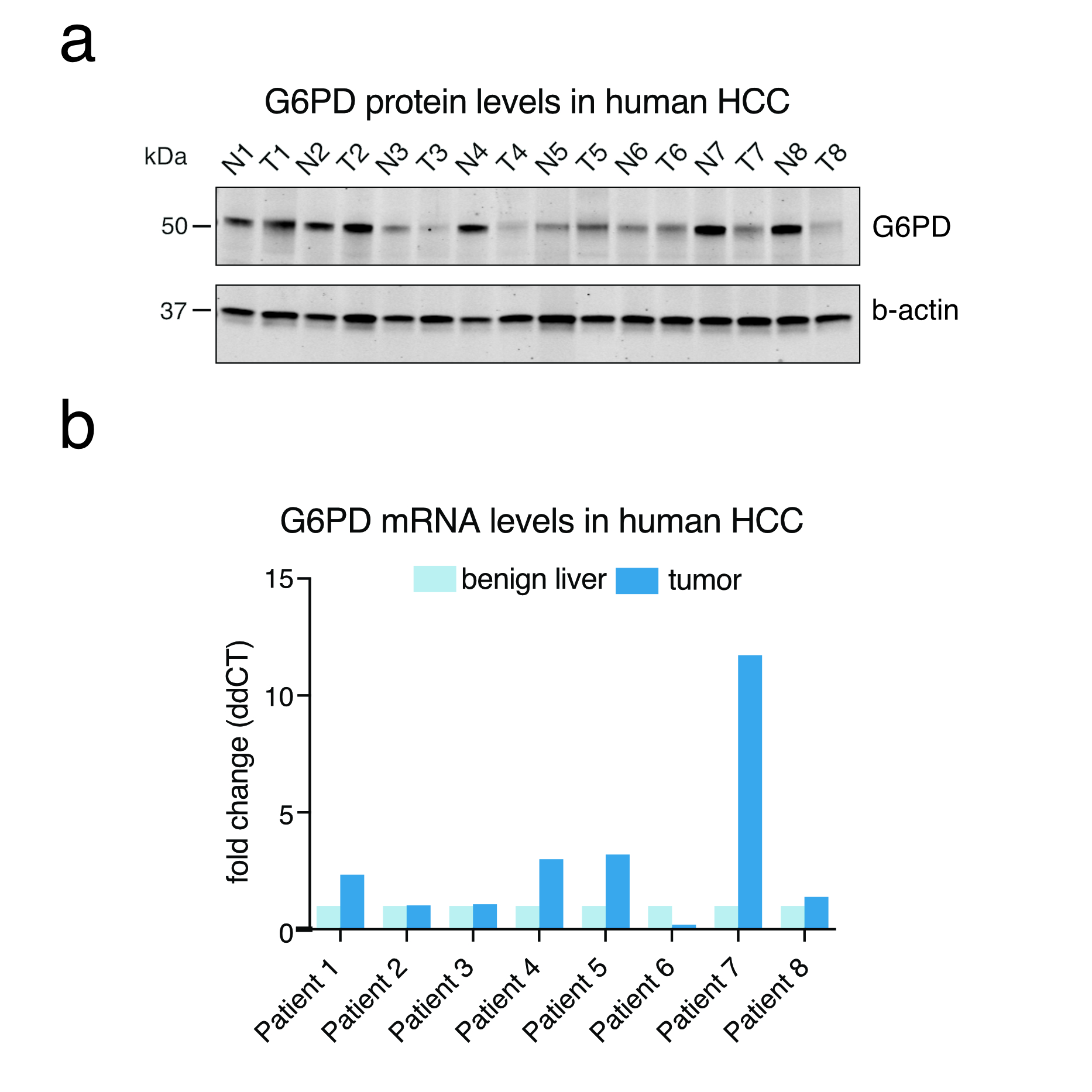


**Supplemental Figure 1: Related to Figure 1. G6PD mRNA levels in paired human HCC samples. (a)** Immunoblot of 8 human benign liver/liver tumor pairs obtained from the Cooperative Human Tissue Network (CHTN). Cropped blot images were obtained from the same gel full blot found at the end of the supplement. **(b)** G6PD transcript levels in the 8 paired benign liver and tumor samples. G6PD mRNA was measured with gene-specific primers using qRTPCR. Relative G6PD mRNA levels were normalized to 18S and calculated using the ddCT method (1).

**Supplemental Figure 2: Related to Figure 2. miR-122 and miR-1 are suppressed in HCC.** Violin plots reveal hsa-miR-122-5p (miR-122) and hsa-miR-1-3p (miR-1) are suppressed in tumors of liver cancer patients as compared to benign liver **(a, b)**. miR-122 and miR-1 expression data (log2(normalized counts+1)) in liver cancer patients was downloaded from The Cancer Genome Atlas using the UCSC Xena Cancer browser (xenabrowser.net).

**Supplemental Figure 3: Related to Figure 3 and 5(2). Validation of G6PD as a miR-122 and miR-1 target.** Luciferase reporter assay. H293-T cells were co-transfected with psi-CHECK2 vector (50ng) harboring full length or mutant 3’-UTR of G6PD and with miR-122 **(a, b)** or miR-1 **(c, d)** using Lipofectamine 3000. Cells were cultured for 48 hours at which point luciferase activity was measured per manufacture’s protocol. Renilla luciferase was normalized to Firefly luciferase control (RLU).


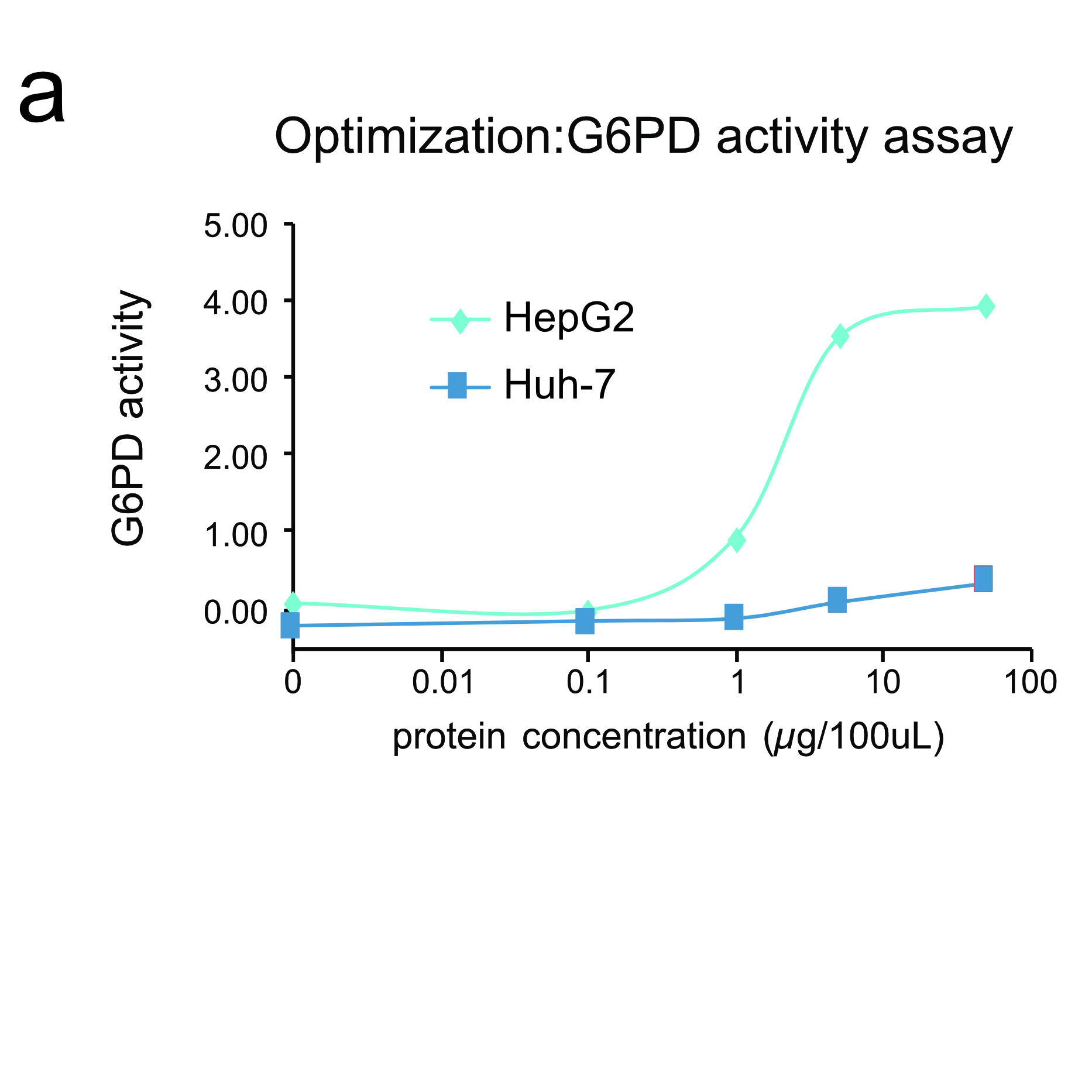


**Supplemental Figure 4: Related to Figure 5. Optimization of G6PD activity assay.** NADPH/NADP levels were used to calculate G6PD activity (3, 4)**.** Briefly, HepG2 and Huh-7 cells were lysed in non-denaturing cell lysis buffer (50mM Tris-HCl-pH7.5, 0.5% Triton-X-100, supplemented with phosphatase inhibitors and PMSF). Protein lysates were serially diluted from 100µg to 0.001µg of protein. Each concentration was added in two duplicate sets to 96-well plate. To the first duplicate set, we added 100ul of Combination Reaction buffer (50mM Tris-HCL-pH8.1, 1mM MgCL2, 0.2mM 6PG, 0.2mM G6P, 0.1mM NADP+), and to the second duplicate set, we added 6PGD Reaction Buffer (50mM Tris-HCL-pH8.1, 1mM MgCL2, 0.2mM 6PG, 0.1mM NADP+). Absorbance of each well was measured at 341nM over several time-points at 37°C. G6PD activity was calculated as the difference in absorbance (NADPH levels) between the wells treated with the Combination reaction buffer and wells treated the 6PGD reaction buffer.

**Supplemental Figure 5: Related to Figure 6. Viability of cells treated with G6PD siRNA.** Huh7 cells were transfected with 75nM of negative control siRNA (NC) or anti-G6PD siRNA (siG6PD) and cultured for 48 hours. Cell viability assays were measured using CellTiter-Glo® Luminescent assay. Cell viability over time was determined as change in luminescence from 0-hour time point.

**Supplement Table S1: Related to Figure 1b.** G6PD is associated increasing tumor grade in liver cancer analyzed using Tukey’s Honest adjustment of means comparison method.

| **Supplement Table 1.**  LIHC-TCGA data related to Figure 1b | | | |
| --- | --- | --- | --- |
| *Comparison of mean G6PD expression in liver cancer with respect to tumor grade* | | | |
| **Tumor Grade Comparison** | | **difference in means** | **adjusted p-value** |
| G1-G2 | 0.6943 | | 0.0115 |
| G2-G3 | 0.5656 | | 0.0057 |
| G3-G4 | 0.2752 | | 0.9238 |
| G1-G3 | 1.260 | | 0.000001 |
| G1-G4 | 1.535 | | 0.00559 |

**Supplement Table S2. Antibodies/Reagents used in the manuscript**

| **Reagent** | **Company** | **Purpose** | **Catalog Number** |
| --- | --- | --- | --- |
| Antibody: G6PD | Abcam | Immunoblot | ab76598 |
| Antibody: ⍺-actinin | Santa Cruz | Immunoblot | Sc-17829 |
| Antibody: Vinculin | Protein-Tech | Immunoblot | 66305-1-Ig |
| Antibody: GAPDH | Cell Signaling | Immunoblot | 5174L |
| SMARTpool: ON-TARGETplus G6PD siRNA | Dharmacon | siRNA experiments | L-008181-02-0010 |
| ON-TARGETplus Non-targeting Control Pool | Dharmacon | siRNA experiments | D-001810-10-20 |
| CellTiter-Glo® Luminescent Cell Viability Assay | Promega | Cell Viability experiments | G7572 |

**Supplement Table S3. Oligos/primers/RNA used in the study**

| **Oligo Name** | **Sequence** | **Purpose** |
| --- | --- | --- |
| h-ACTB-RT-F | 5'- CTGGCACCACACCTTCTACAATG -3' | qPCR |
| h-ACTB-RT-R | 5'- TAGCACAGCCTGGATAGCAACG -3’ | qPCR |
| h-GAPDH-RT-F | 5'-TCCTGCACCACCAACTGCTTAG-3' | qPCR |
| h-GAPDH-RT-R | 5'-TGCTTCACCACCTTCTTGATGTC-3' | qPCR |
| h-G6PD-RT-F | 5’-TGCCTTCCATCAGTCGGATACAC-3’ | qPCR |
| h-G6PD-RT-R | 5’ACTCGTGAATGTTCTTGGTGACG-3’ | qPCR |

**Supplemental Images: Raw images of blots used in our study**

**Blot 1: Related to Supplement Figure 1a**


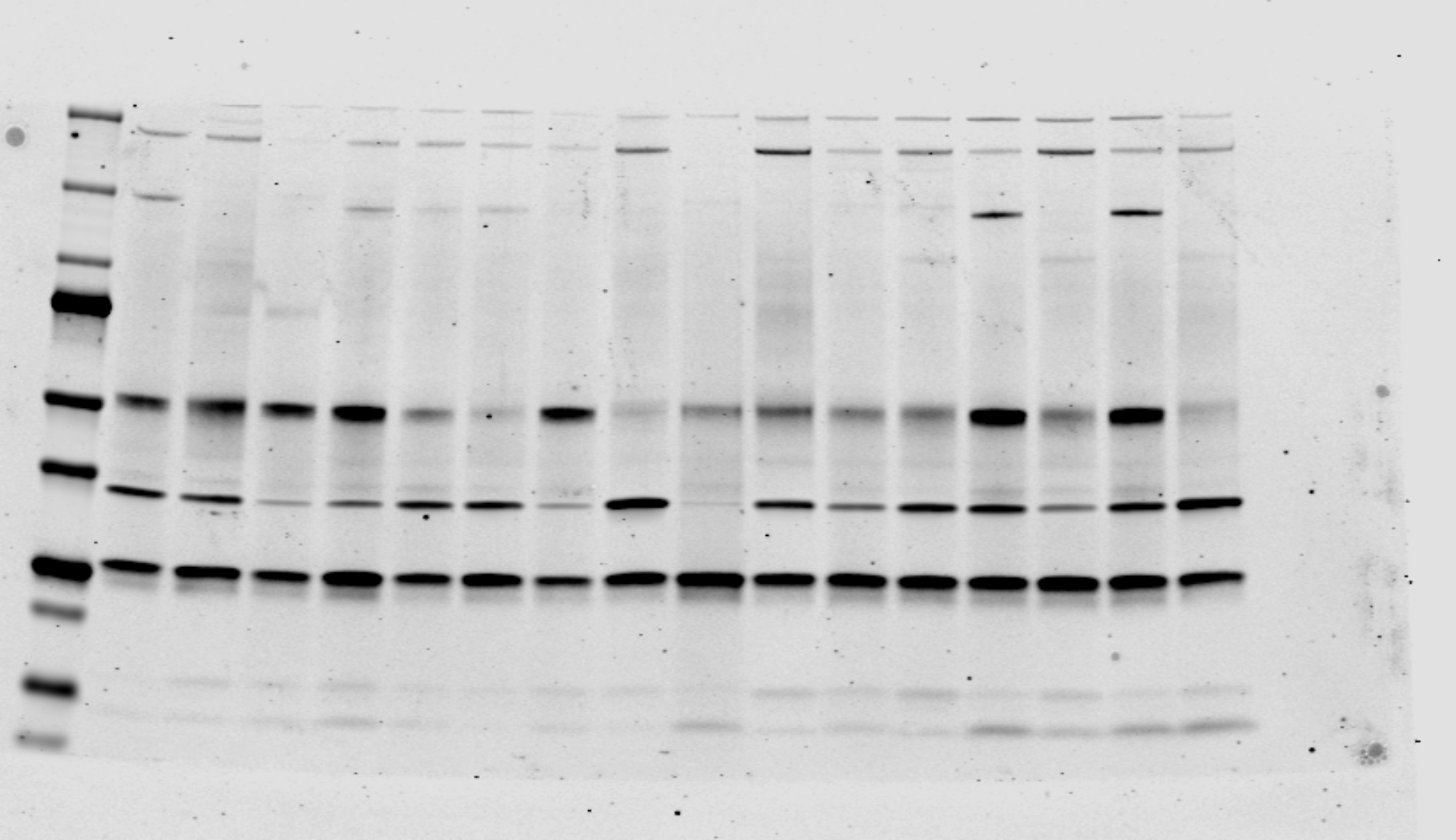


**Blots Related to Figure 4b**


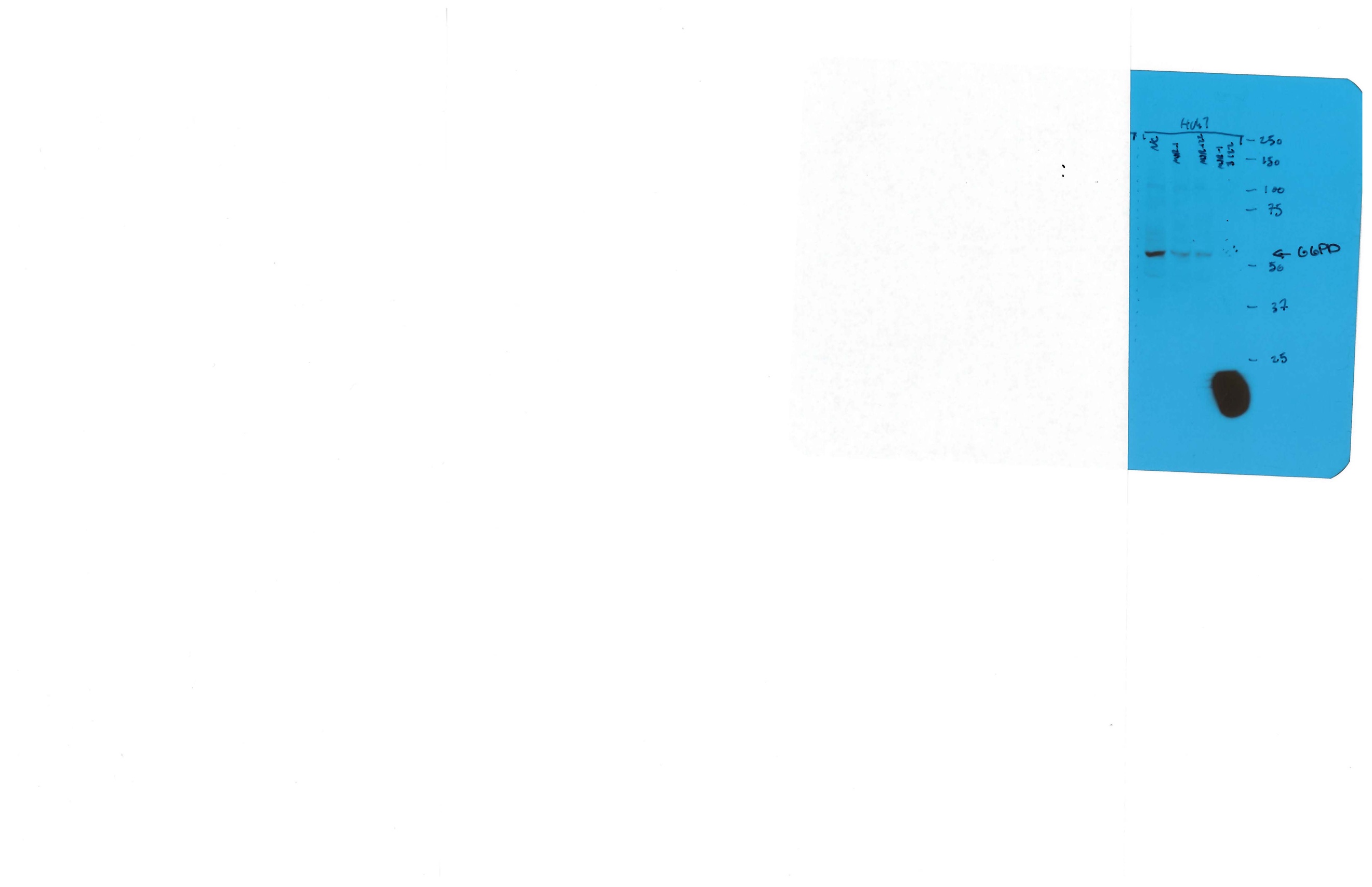


Huh7 cells, G6PD, ECL, 2 min. exposure


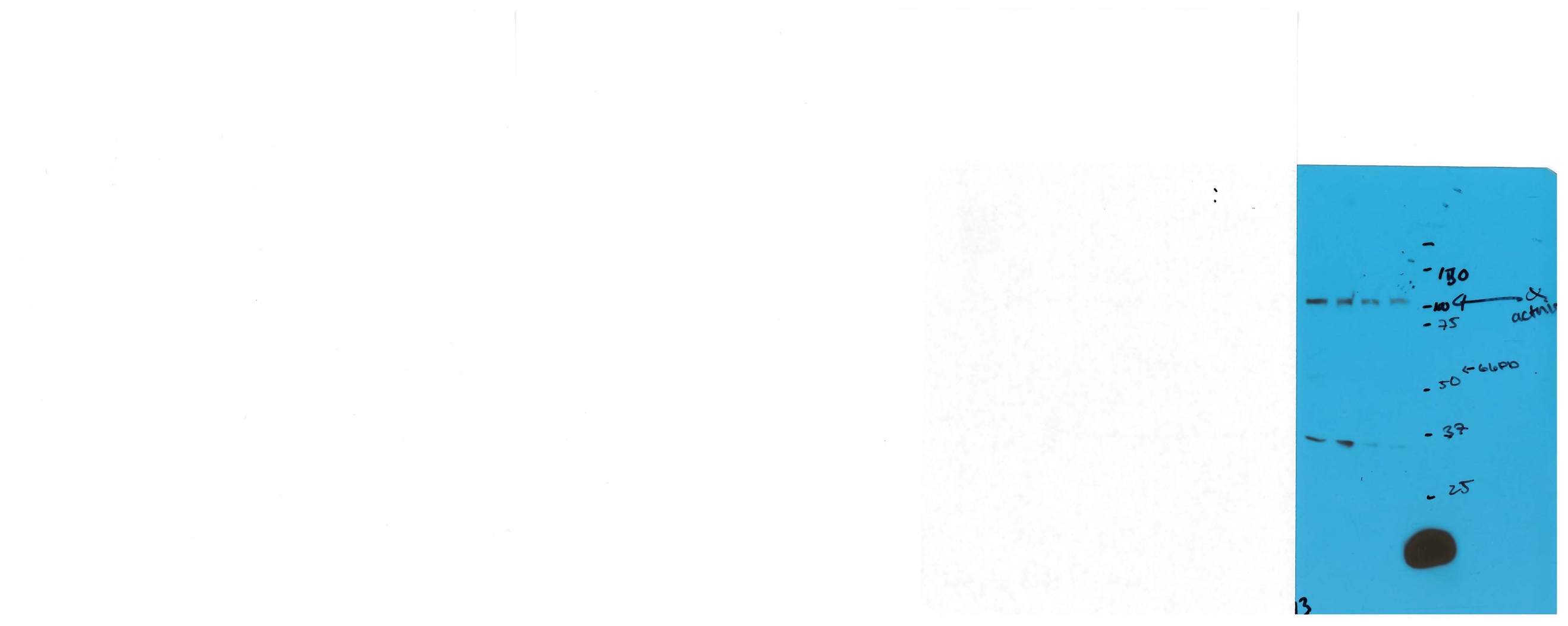


Huh7 cells, a-actinin, ECL, 2 min exposure


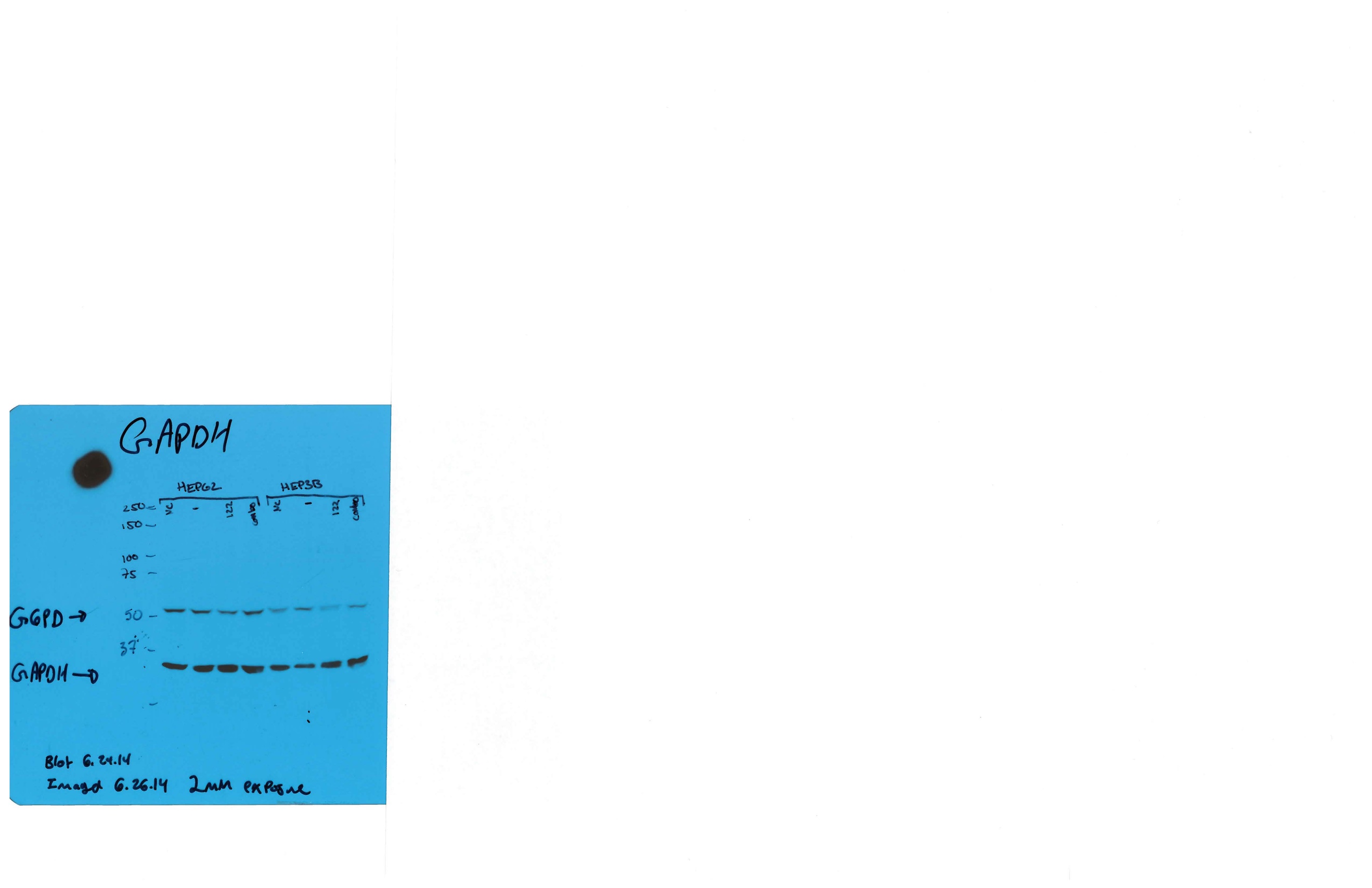


HepG2, Hep3B cells, G6PD, ECL, 2 min exposure


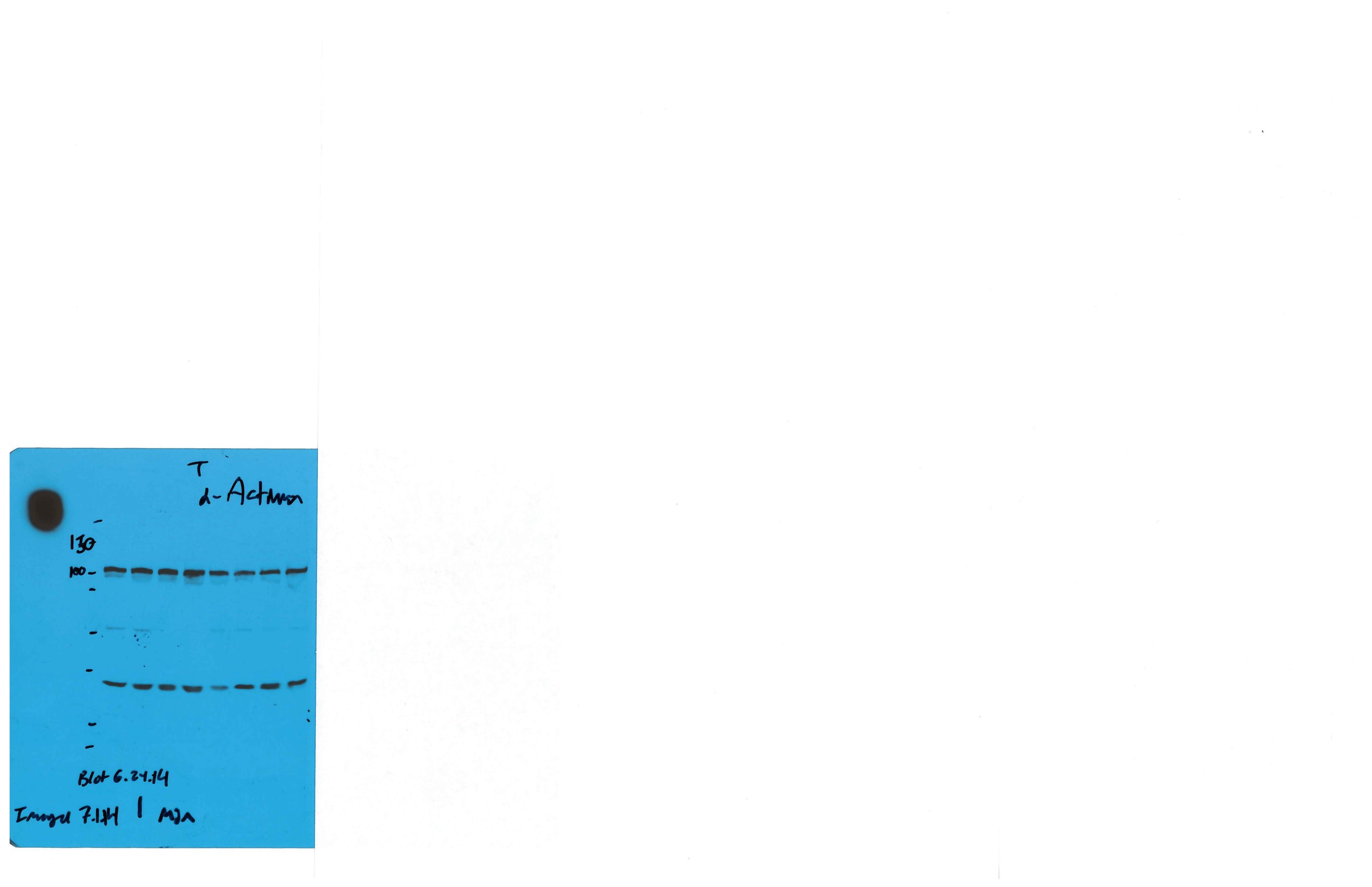


HepG2, Hep3B cells, a-actinin, ECL, 2 min exposure

**Blot 3: Related to Figure 6a**


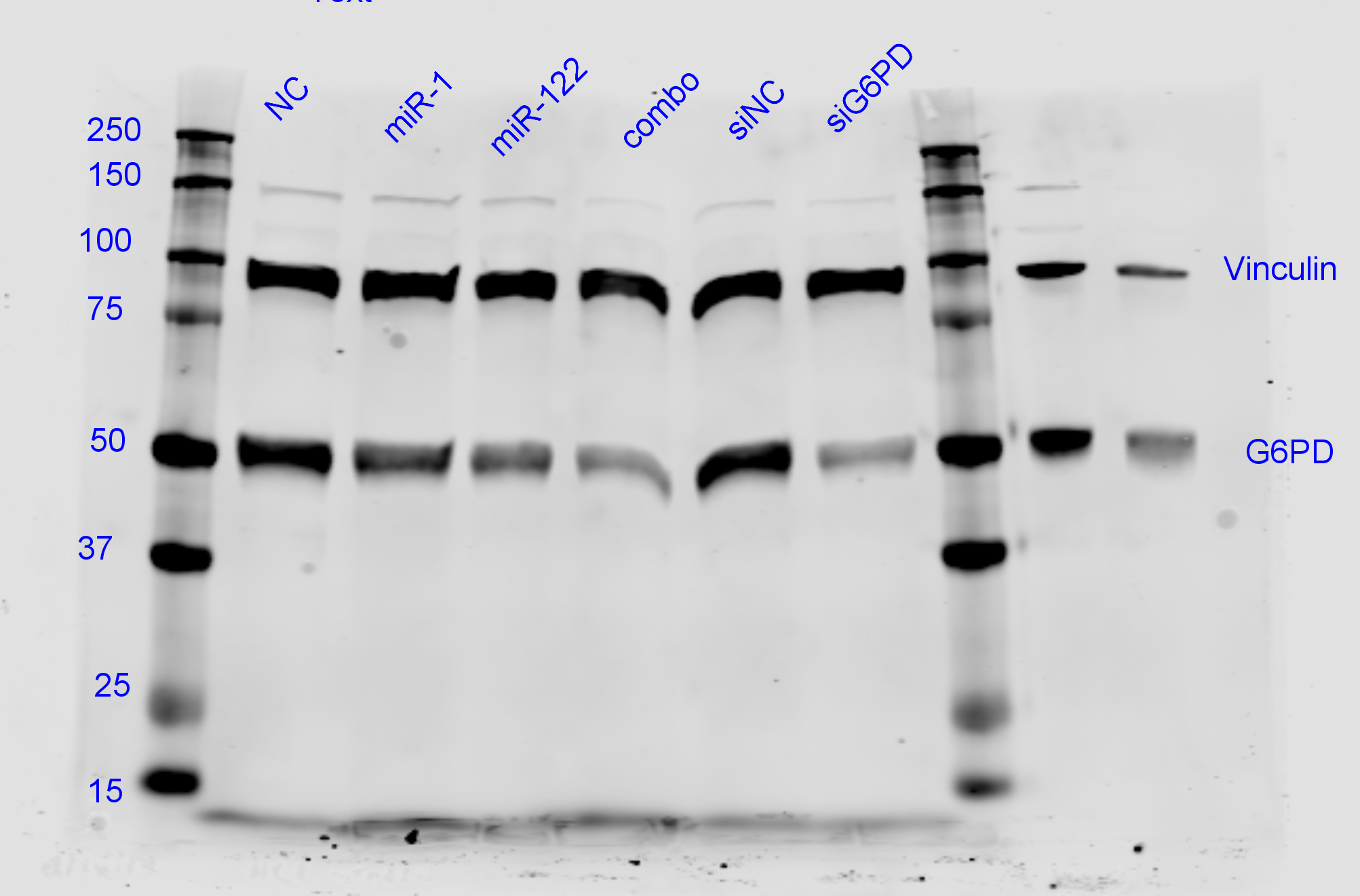


**References:**

1) Livak KJ, Schmittgen TD. Analysis of relative gene expression data using real-time quantitative PCR and the 2(-Delta Delta C(T)) Method. *Methods* 2001; **25**: 402-8.

2) Coulouarn C, Factor VM, Andersen JB, Durkin ME, Thorgeirsson SS. Loss of miR-122 expression in liver cancer correlates with suppression of the hepatic phenotype and gain of metastatic properties. *Oncogene* 2009; **28**: 3526-36.

3) Tian WN, Braunstein LD, Apse K, Pang J, Rose M, Tian X, et al. Importance of glucose-6-phosphate dehydrogenase activity in cell death. *Am J Physiol* 1999; **276**: C1121-31.

4) Tian WN, Pignatare JN, Stanton RC. Signal transduction proteins that associate with the platelet-derived growth factor (PDGF) receptor mediate the PDGF-induced release of glucose-6-phosphate dehydrogenase from permeabilized cells. *J Biol Chem* 1994; **269**: 14798-805.
